# HIF-1α and HDAC1 mediated regulation of FAM99A-miR92a signaling contributes to hypoxia induced HCC metastasis

**DOI:** 10.1038/s41392-020-00223-6

**Published:** 2020-07-07

**Authors:** Bixing Zhao, Kun Ke, Yingchao Wang, Fei Wang, Yingjun Shi, Xiaoyuan Zheng, Xiaoyu Yang, Xiaolong Liu, Jingfeng Liu

**Affiliations:** 1 The United Innovation of Mengchao Hepatobiliary Technology Key Laboratory of Fujian Province, Mengchao Hepatobiliary Hospital of Fujian Medical University, Fuzhou, China; 2 The First Affiliated Hospital of Fujian Medical University, Fuzhou, China; 3 The School of Basic Medical Sciences, Fujian Medical University, Fuzhou, China

**Keywords:** Metastasis, Cancer microenvironment

**Dear Editor**,

Hypoxic microenvironment is clinically associated with metastasis and poor prognosis of numerous cancers. Previous studies have shown that many protein-coding genes and microRNAs are regulated upon hypoxia and involved in the progression of cancer. However, the roles of lncRNAs in the hypoxia-responsive gene networks and how lncRNA-related signaling network regulates hypoxia induced tumor metastasis is still not clear.

To identify potential key lncRNAs associated with HCC, lncRNA sequencing analysis was performed in 61 paired HCC tissues and corresponding para-tumor tissues. Among the differentially expressed lncRNAs (Supplementary Fig. S1), FAM99A was found to be downregulated by more than sevenfolds (Supplementary Fig. S2). RT-qPCR validated the downregulation of FAM99A in another cohort of 103 HCC tissues (Fig. 1a). In addition, the downregulation of FAM99A was also observed in the TCGA dataset (Supplementary Fig. S3). Further clinical pathology data analysis shows that the decreased expression of FAM99A significantly correlated with tumor capsule, differentiation, and recurrence (Supplementary Table S1) and poor prognosis (Fig. 1b). Univariate and Multivariate Cox regression analysis demonstrates that FAM99A low expression served as an independent prognostic factor for overall survival of HCC patients (Supplementary Table S2).

**Fig. 1:**
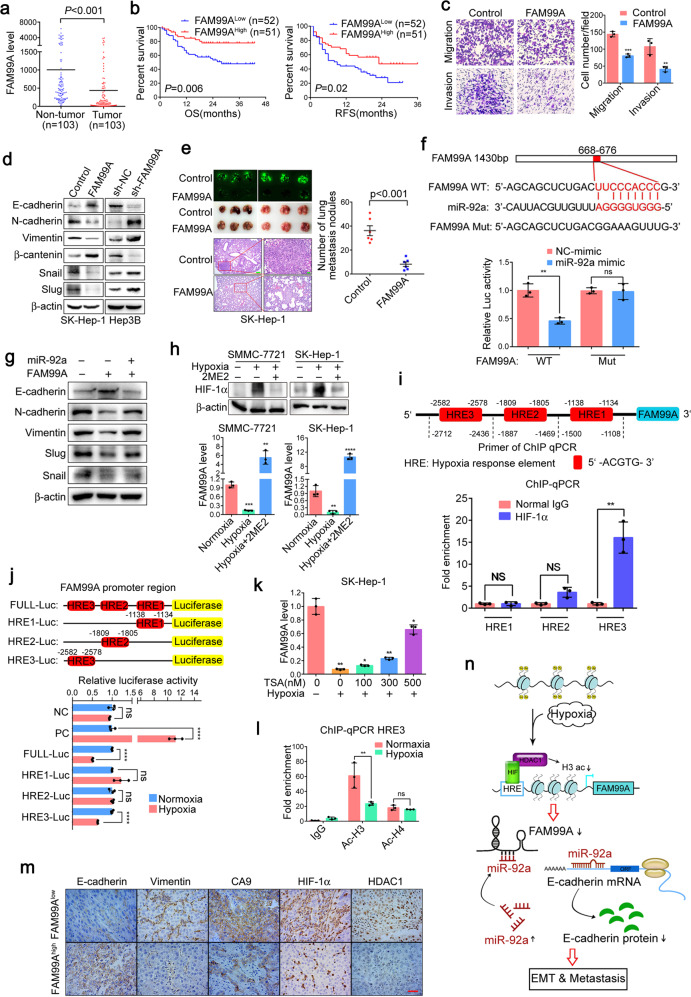
**a** RT-qPCR validation of FAM99A expression in 103 HCC patients with paired tumor and para-tumor tissues. 18S rRNA was used as an internal control. Student’s *t*-test was used to compare the difference between para-tumor and tumor tissues. **b** Kaplan–Meier analysis revealed that low expression of FAM99A significantly associated with shorter OS (overall survival) and RFS (recurrence free survival). The levels of FAM99A were analyzed using real-time qRT-PCR, and the median value of all 103 cases was chose as the cutoff point for separating the FAM99A-low expression and FAM99A-high expression groups. Log-rank test was used to assess the statistical difference. **c** Representative and quantification results of transwell cell migration assay and invasion assay in SK-Hep-1 cells stably transfected with FAM99A or pCDH control. **d** Western blotting detection of epithelial (E-cadherin and β-catenin), mesenchymal (N-cadherin and Vimentin) and transcription factors (Slug and Snail) in SK-Hep-1 cells with FAM99A overexpression or Hep3B cells with FAM99A knockdown, respectively. **e** Representative images and quantification data of metastatic nodules in the lung tissues of mice. Stable cell lines of FAM99A overexpression or control were injected by tail vein in B-NDG mice. Upper left: macroscopic and fluorescent images. Upper right: quantification of lung metastatic nodules. Lower: representative images of lung metastatic nodules stained with H&E (magnification, ×50 and ×200; scale bar, 100 and 20 μm). **f** Up: prediction of miR-92a binding sites in FAM99A (FAM99A-WT) and the design of FAM99A mutation sequence (FAM99A-Mut). down: luciferase reporter assays were performed in 293T cells co-transfected with the miR-92a mimic and FAM99A-WT or FAM99A-Mut reporter plasmid. **g** Western blotting analysis of EMT-associated markers in SK-Hep-1 cells stably transfected with FAM99A or co-transfected with miR-92a and FAM99A. **h** qRT-PCR analyzing the expression of FAM99A when treated with or without hypoxia inhibitor (2-ME2) under hypoxic conditions. **i** Up: The schematic illustration of HRE regions in FAM99A promoter and the primers of ChIP quantitative PCR (ChIP-qPCR). Down: ChIP-qPCR assay was performed to identify the binding region between HREs of FAM99A promoter and HIF-1α. **j** Up: the schematic illustration of luciferase reporter gene plasmid. Down: Luciferase reporter gene assays were performed to compare the luciferase activity of HREs or full length of FAM99A promoter under normal oxygen or hypoxic conditions; PC, positive control **k** qRT-PCR analysis of FAM99A expression in SK-Hep-1 cells cultured with or without different concentrations of TSA for 24 h under normoxic or hypoxic conditions. **l** ChIP-qPCR analysis was conducted on the HRE3 using anti-acetyl-histone H3 and H4 under normoxic or hypoxic conditions. **m** Representative images of IHC staining in human HCC tissues (magnification, ×200; scale bar, 20 μm). **n** Schematic overview of HIF-1α and HDAC1 mediated regulation of FAM99A-miR92a signaling contributes to hypoxia induced hepatocellular carcinoma metastasis. Error bars symbolized standard deviation acquired from three independent experiments and all the data were shown as mean ± SD, *P < 0.05; **P < 0.01; ***P < 0.001; ****P < 0.0001

The next cellular phenotype studies show that FAM99A overexpression inhibited migration and invasion of SK-Hep-1 cells (Fig. 1c and Supplementary Fig. S6), while FAM99A knockdown promoted migration and invasion of Hep3B cells (Supplementary Figs. S5, S6), both in transwell and wound-healing assay.

Whether FAM99A can regulate EMT in HCC cells was further investigated. As shown in Fig. 1d, FAM99A increased the expression of epithelial markers E-cadherin and β-catenin, but decreased the E-cadherin repressor snail and slug as well as mesenchymal markers N-cadherin and Vimentin in SK-Hep-1 cells. Conversely, FAM99A knockdown in Hep3B cells induced a reverse trend (Fig. 1d).

The in vivo tail vein injection mouse model shows that FAM99A dramatically inhibited HCC lung metastasis of both SK-Hep-1 and SMMC-7721 cells in mouse model (Fig. 1e and Supplementary Fig. S7). Furthermore, FAM99A over-expression significantly prolonged the survival of tumor bearing mice (Supplementary Fig. S8).

To explore the molecular mechanisms by which FAM99A exerted its functions in HCC, bioinformatics analysis was carried out by using TargetScan, miRanda and RNAhybrid, we found that miR-92a which may promote tumor metastasis via E-cadherin^1^ was a potential target of FAM99A (Fig. 1f). Luciferase reporter assay shows that transfection with miR-92a significantly inhibited the luciferase activity in 293T cells, while miR-92a mimic failed to regulate the luciferase activity when the binding site was mutated (Fig. 1f), which indicated that miR-92a directly bond to FAM99A. Moreover, FAM99A over-expression significantly decreased the expression of miR-92a (Supplementary Fig. S9). Furthermore, we also found the significantly increased miR-92a level in HCC tissues comparing to adjacent para-tumor tissues (Supplementary Fig. S10). Besides, qRT-PCR assay shows that there was a negative correlation between the expression of FAM99A and miR-92a in HCC tissues (*n* = 53) (Supplementary Fig. S11).

Moreover, the effect of FAM99A on HCC cells migration and invasion was rescued by co-transfection of miR-92a (Supplementary Figs. S12 and S13). Meanwhile, the change of EMT- related protein expression induced by FAM99A was also abrogated by miR-92a (Fig. 1g).

Recent studies have shown that aberrant expression of some lncRNAs was attributed to hypoxic microenvironment of cancers,^2,3^ we wonder if the decreased expression of FAM99A in HCC was regulated by hypoxia. As shown in Figs. S14 and S15, both hypoxia and CoCl_2_ treatment decreased the expression of FAM99A, and the hypoxia induced FAM99A downregulation could be inhibited by HIF-1 inhibitor 2-ME2 (Fig. 1h), indicating that hypoxia reduced FAM99A expression was in a HIF-1α dependent manner. To understand the underlying mechanism of FAM99A downregulation under hypoxia, we surveyed the promoter region of the FAM99A and identified 3 hypoxia response elements (HREs) (Fig. 1i). The ChIP assay only shows enrichment of the fragment containing HRE3 (−2712 to −2436 bp) in HIF-1α-immunoprecipitated chromatin (Fig. 1i). More importantly, hypoxia could dramatically increase the binding of HIF-1α to FAM99A promoter (Supplementary Fig. S16). The luciferase analysis confirms that hypoxia significantly inhibited full-length and HRE3 of FAM99A promoter activity while without affect the HRE1 or HRE2 activity of FAM99A promoter in 293T cells (Fig. 1j). The TCGA data analysis also shows that there was a significant negative correlation between FAM99A and HIF-1α expression in HCC (Supplementary Fig. S17). Taken together, these data suggest that FAM99A is transcriptionally inhibited by HIF-1α during hypoxia.

HDAC1 interacted with HIF-1α to downregulate the expression of HIF-1α downstream target genes.^4^ Therefore, we hypothesized that hypoxia induced FAM99A downregulation was depending on HDAC1 and its mediated histone deacetylation. First, we found that HDAC1 but not HDAC3 negatively regulated FAM99A expression (Supplementary Figs. S18 and S19). Moreover, the HDAC inhibitor TSA increased the expression of FAM99A in a dose dependent manner (Supplementary Fig. S20), and the suppression of FAM99A by hypoxia was impaired at the presence of TSA (Fig. 1k and Supplementary Fig. S21). The data from TCGA further confirmed a significant negative correlation between HDAC1 and FAM99A expression while no correlation between HDAC3 and FAM99A in HCC tissues (Supplementary Fig. S22). ChIP assay also showed a significant interaction between HDAC1 and FAM99A promoter (Supplementary Fig. S23); more importantly, hypoxia promoted the HDAC1 binding to FAM99A promoter region (Supplementary Fig. S23).

Next, we further investigate whether HDAC1 mediated histone deacetylation is involved in the negative regulation of FAM99A by hypoxia. ChIP assay showed the enrichment of total histone H3 acetylation but not total H4 acetylation in FAM99A promoter region which contain HRE3 (Supplementary Fig. S24). More importantly, hypoxia significantly impaired enrichment of histone H3 acetylation in this promoter region (Fig. 1l).

Hypoxia has been shown to contribute to metastasis of various cancers including HCC. To explore the role of FAM99A in hypoxia induced HIF-1α-mediated metastasis, transwell assay shows that overexpression of FAM99A significantly attenuated the hypoxia induced HCC cell migration and invasion (Supplementary Fig. S25). Moreover, 2ME2 further inhibited the migration and invasion activity of HCC cells (Supplementary Fig. S25). In addition, immunohistochemical staining in HCC tissue samples showed that the expression of E-cadherin was significantly higher while the expression of Vimentin, HDAC1, HIF-1α, and another hypoxia marker CA9 were relatively lower in the FAM99A^high^ group than the FAM99A^low^ group (Fig. 1m). In the mouse HCC nodules, the same expression trend was observed (Supplementary Fig. S26).

In our study, we show evidence that FAM99A negatively regulated EMT via miR-92a, which may regulate EMT via targeting E-cadherin.^1^ In addition to E-cadherin, miR-92a may also regulate EMT via other indirect targets, such as PTEN.^5^ As the expression of other EMT markers was also influenced by miR-92a mimic (Fig. 1g), it suggested that indirect targets such as PTEN might participate in the regulation of EMT. Therefore, the downstream signaling network of FAM99A in regulating EMT still needs further investigation.

In conclusion, our finding suggests that HIF-1α and HDAC1 transcriptionally inhibit FAM99A expression through histone H3 deacetylation during hypoxia. Then, FAM99A inhibits HCC metastasis and EMT by negatively regulating miR-92a, thus hypoxia microenvironment contributes to HCC progression via HIF-1α/HDAC1/FAM99A/miR-92a axis (Fig. 1n). Our study highlights the relationship between epigenetic regulation and lncRNAs, and demonstrates that FAM99A is involved in hypoxia signal transduction in HCC, which is a critical event in tumor progression.

## Supplementary information

Supplementary information

## Data Availability

The data sets used and/or analyzed during the current study are available from the corresponding author on reasonable request.
