# Supplementary material for: HIF-1α and HDAC1 mediated regulation of FAM99A-miR92a signaling contributes to hypoxia induced HCC metastasis
Source: Signal Transduct Target Ther. 2020 Jul 7;5:118. doi: 10.1038/s41392-020-00223-6 (PMC7341733; doi:10.1038/s41392-020-00223-6)
Supplement: Supplementary file 1 — Supplementary information [file 41392_2020_223_MOESM1_ESM.docx]

Supplementary Materials for

HIF-1α and HDAC1 mediated regulation of FAM99A-miR92a signaling contributes to hypoxia induced HCC metastasis

Bixing Zhao^1^, Kun Ke^1,2^, Yingchao Wang^1^, Fei Wang^1^, Yingjun Shi^1^, Xiaoyuan Zheng^1^, Xiaoyu Yang^3^, Xiaolong Liu^1^, Jingfeng Liu^1,2^

*^1^The United Innovation of Mengchao Hepatobiliary Technology Key Laboratory of Fujian Province, Mengchao Hepatobiliary Hospital of Fujian Medical University, Fuzhou, China*

*^2^The First Affiliated Hospital of Fujian Medical University, Fuzhou, China*

*^3^The School of Basic Medical Sciences, Fujian Medical University, Fuzhou, China*

*These authors contributed equally to this work: Bixing Zhao, Kun Ke, Yingchao Wang, Fei Wang*

*Correspondence: Xiaolong Liu (*[*xiaoloong.liu@gmail.com*](mailto:xiaoloong.liu@gmail.com)*) or Jingfeng Liu (*[*drjingfeng@126.com*](mailto:drjingfeng@126.com)*)*

**This PDF file includes:**

Materials and Methods

Supplementary Text

Figures. S1 to S26

Tables S1 to S3

Materials and Methods

**Patients and specimens**

Clinical samples for RNA transcriptome sequencing and real-time qPCR were obtained from hepatocellular carcinoma (HCC) patients at Mengchao Hepatobiliary Hospital of Fujian Medical University. All included patients were diagnosed as HCC by pathological examination and none of them received any anti-tumor therapy before hepatectomy. The paired tumor and para-tumor tissues were collected and stored at -80℃ after surgery. Specimen collection and usage were approved by the Institutional Review Board (IRB) of Mengchao Hepatobiliary of Fujian Medical University. Meanwhile, informed consents were signed by the patients.

**RNA extraction, reverse transcription, and real-time quantitative PCR**

Total RNA was extracted from HCC tissue samples or cells using TransZol Up RNA kit (TransGen, Beijing, China) according to the manufacturer’s instructions. The quality and concentration of RNA were measured by the NanoDropND-2000 (Thermo Scientific, IL, USA). After that, 1μg RNA was reverse transcribed into cDNA following the Transcriptor Frist Strand cDNA Synthesis Kit (Roche, Basel, Switzerland). The quantitative PCR reaction was conducted by the StepOne Plus^TM^ real-time PCR system (AB Applied Biosystems, CA, USA) using the agent of Bsetar® SybrGreen qPCR Mastermix (DBI, Ludwigshafen, Germany) following the manufacturer’s instructions. For normalization, human 18S ribosome RNA was used as endogenous control. All primers used in the study were listed in Supplementary Table S3. The relative expression level of genes was calculated by the 2^−ΔΔCt^ method.

**Cell culture and hypoxia treatment**

The human hepatoma cell lines Hep3B, SK-Hep-1 and human embryonic kidney cell HEK 293T were purchased from the American Type Culture Collection (ATCC, VA, USA). The SMMC-7721 cell line was obtained from the Chinese Academy of Science (Shanghai, China). The lines were verified by short tandem repeats analysis (STR). All cell lines were cultured in Dulbecco’s modified Eagle’s medium (DMEM, Gibco, CA, USA) supplemented with 10% fetal bovine serum (FBS, Excel, Australia),100 IU penicillin, and 100 mg/mL streptomycin (Bio Basic) and incubated with 5% CO_2_ at 37℃. For hypoxia treatment, cells were cultured in a three-gas incubator (Thermo Scientific, MA, USA) with 1% O_2_, 5% CO_2_ and 94% N_2_ or treated with 100 μmol/L cobalt chloride (CoCl_2_, Sigma-Aldrich, MO, USA) for 24h.

**Plasmid construction and stable cell line establishment**

To observe the effects of FAM99A overexpression or knockdown on the function of hepatocellular carcinoma cells in vitro and in vivo, we constructed lentivirus plasmids: FAM99A overexpression (FAM99A) and blank vector (Control), FAM99A knockdown plasmid (sh-FAM99A) and negative control plasmid (sh-NC). All plasmids were then packaged into lentiviruses *via* lipofectamine 3000 (Invitrogen, NY, USA). SK-Hep-1 cell with low endogenous expression of FAM99A and SMMC-7721 with high endogenous expression of FAM99A were infected with overexpression and knockdown lentivirus, respectively. Then, stable cell lines were established by the selection of puromycin (3μg/mL) for 2 weeks. HDAC1 (#13820) and HDAC3 (#13819) plasmids were obtained from Addgene.

**siRNA transfection and shRNA construction**

siRNA and negative control were purchased from GenePharma (Shanghai, China). The corresponding sequences were as follows: siFAM99A (sense, 5’-CCCACUGUGGGAAUCCUAUTT-3’ and antisense, 5’-AUAGGAUUCCCACAGUGGGTT-3’), siHDAC1 (sense, 5’-GCCGGUCAUGUCCAAAGUATT-3’ and antisense, 5’- UACUUUGGACAUGACCGGCTT-3’), siHDAC3 (sense, 5’-UCGCCUGGCAUUGACCCAUTT-3’ and antisense, 5’- AUGGGUCAAUGCCAGGCGATT-3’), siNC (sense, 5’-UUCUCCGAACGUGUCACGUTT-3’ and antisense, 5’-ACGUGACACGUUCGGAGAATT-3’). 293T or hepatoma cells were cultured with 6-well plates the day before. Then, 293T or hepatoma cells were transfected with 100uM siRNA, 125ul Opti-MEM medium and 6ul lipofectamine 3000 (Invitrogen, CA, USA). The pGreenPuroTM vector (Cat. #SI505A-1) for shRNA construction was purchased from System Biosciences (SBI, CA, USA). The design and construction of shRNA following the user manual of SBI.

***In vitro* cell migration, invasion, and wound healing assay**

Transwell filter insert (Corning-Costar, NY, USA) containing 8 μm polycarbonate membrane and wound healing culture insert (ibidi, Gräfelfing, Germany) were used for evaluating the migration ability of hepatoma cells *in vitro*. Briefly, 5×10^4^ SK-Hep-1 or 8×10^4^ Hep3B cells resuspended in 200 μl DMEM or MEM medium without serum were seeded to the upper chamber, and 600 μl DMEM or MEM medium supplemented with 10% FBS was added to the bottom chamber. Cells were cultured at 37℃ for 24h or 48h, respectively. For invasion assay, the method was similarly to migration, but cells were seeded to the top chamber coated with Matrigel (Corning-Costar, NY, USA).

 For the wound healing assay, prepare the cell suspension of a 3-7×10^5^ cells/ml in serum-free medium and apply 70 μl of cells each well into the culture insert (ibidi, Gräfelfing, Germany). After cell adherence, inserts were removed and microscopic observation at different time points was performed until cells near confluence.

***In vivo* metastasis assay**

Four-week-old male B-NDG mice were purchased from Wushi Animal Center (Fuzhou, China) and raised one week under the environment of specific pathogen animal (SPF) grade before the experiment. Then,1×10^6^ SK-Hep-1 cells stably expressing FAM99A and Control or SMMC-7721 cells stably expressing sh-FAM99A and sh-NC that suspended in 100 μl PBS were injected into mice by lateral tail veil (n=6 per group). After 6 weeks, all mice were sacrificed and their lung and liver metastasis tumor were dissected. Then, parts of tumor tissues were embedded in paraffin for H&E and immunohistochemistry (IHC) staining.

**Luciferase assay**

1×10^5^ 293T cells were seeded into 24 well plates in triplicate before transfection. pMIR-REORT luciferase reporter plasmid containing a bind region of FAM99A (FAM99A-WT) or a mutated fragment (FAM99A-Mut) was co-transfected with miR-92a mimics or control mimics and internal control plasmid β-gal. After 24-hour cultivation, the luciferase activity was calculated by deducting the activity of corresponding control vector β-gal.

To investigate whether FAM99A is transcriptionally regulated by HIF-1α, the luciferase reporter plasmids of pGL2-Basic containing full length of FAM99A promoter or different HREs of FAM99A promoter were co-transfected with internal control plasmid β-gal. Cells were cultured under normal oxygen or hypoxic conditions. After a 24-hour incubation, luciferase activity was measured as above.

**Chromatin immunoprecipitation (ChIP) assay**

ChIP was performed according to the instruction manual of EZ-CHIP^TM^ Chromatin Immunoprecipitation Kit (Millipore, MA, USA). SMMC-7721 cells were cultured in normoxic or hypoxic conditions (1% O_2_) for 24h. Subsequently, the cells were crosslinked with formaldehyde and sonicated to an average size of 300-500bp. Lysates were immunoprecipitated with anti-Acetyl-Histone H3 (Millipore, MA, USA), anti-Acetyl-Histone H4 (Millipore, MA, USA), anti-RNA polymerase II (Epigentek, NY, USA) or normal rabbit IgG (Millipore, MA, USA). Finally, the protein or DNA complexes were reversely crosslinked to free DNA and then later further purified using DNA Spin Columns. The eluted DNA was detected by real-time qPCR.

**Western blotting**

Total protein was extracted from cells using RIPA cell lysis buffer containing proteinase inhibitor. Protein concentration was analyzed by BCA method. Then, 50 μg proteins were western bolted according to the protocol. Antibodies used in this study were as follows: anti-E-cadherin (1:2000; Cell Signaling Technology), anti-N-cadherin (1:2000; Cell Signaling Technology), anti-Vimentin (1:2000; Cell Signaling Technology), anti-slug (1:2000; Cell Signaling Technology), anti-snail (1:2000; Cell Signaling Technology), anti-β-catenin (1:2000; Cell Signaling Technology), anti-β-Actin (1:20000; Abcam), anti-HIF-1α (1:2000; Abcam), anti-HDAC1 (1:2000; Abcam), anti-HDAC3 (1:2000; Abcam), anti-Histone H3 (1:2000; Abways technology,), anti-Histone H4 (1:2000; Abcam), anti-acetyl-Histone H3 (1:2000; Millipore), anti-acetyl-Histone H4 (1:2000; Millipore).

**Immunohistochemistry (IHC)**

Paraffin-embedded sections of hepatocellular carcinoma tissues underwent dewaxing and hydration, antigen retrieval. Then, endogenous peroxidase was blocked by 3% H_2_O_2_. Tissue sections were incubated with E-cadherin (1:400, Cell Signaling Technology, MA, USA), N-cadherin (1:400, Cell Signaling Technology, MA, USA), Vimentin (1:400, Cell Signaling Technology, MA, USA), HIF-1α (1:100, Abcam, Cambridge, UK) and HDAC1 (1:100, Abcam, Cambridge, UK) antibody at 4℃ overnight, following by the second antibody at room temperature for an hour. Finally, tissues were stained by DAB and hematoxylin and then were analyzed by microscope at 400× (Zeiss, Germany).

**Statistical analysis**

Statistical analysis was performed using the software of SPSS 19.0 (SPSS, Chicago, USA) and GraphPad Prism 6.0 (GraphPad Software, San Diego, USA). The difference of FAM99A between HCC and para-tumor tissue was compared by paired Student’s t-test. Person chi-square test was applied to comparing the correlations between FAM99A and clinicopathologic parameters. The Kaplan-Meier survival analysis and log-rank test were used for assessing overall survival and relapse-free survival. Univariable and multivariable Cox proportional hazards regression were performed to analyze the prognostic factors. P <0.05 was considered as statistically significant.


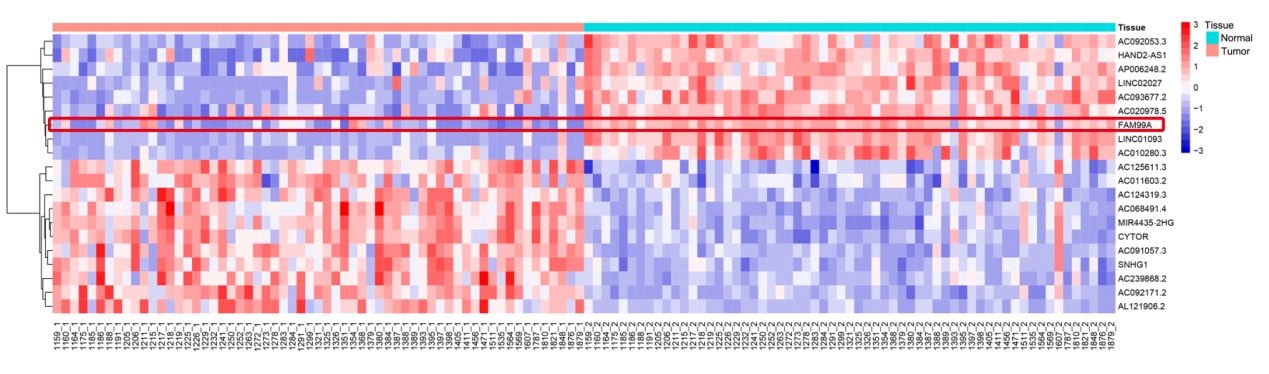


**Figure S1.** Heatmap of the top 20 signature lncRNAs in 61 paired HCC tumor and adjacent para-tumor tissues. The color in the heatmap represents the expression level of lncRNAs: red, up-regulation; blue, down-regulation.

**
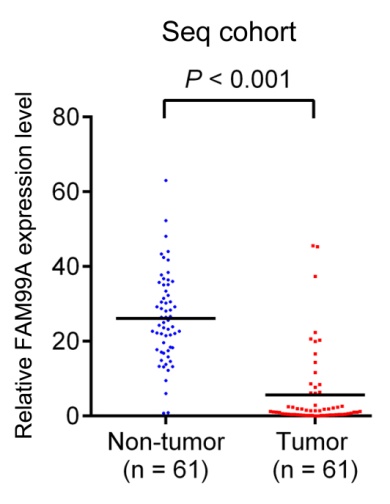
**

**Figure S2.** Transcriptome sequencing detection of FAM99A expression in the cohort of 61 HCC patients with paired tumor and para-tumor tissues.

**
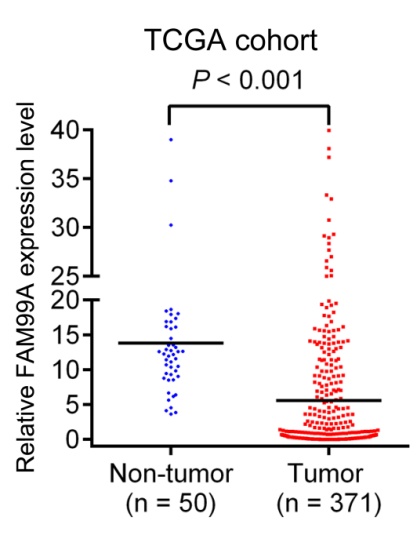
**

**Figure S3.** Relative expression level of FAM99A in adjacent para-tumor tissues (n=50) and tumor tissues (n=371) from TCGA cohort.







**Figure S4.** Expression of FAM99A in SK-Hep-1 cells stably transfected with FAM99A or Control and in Hep3B cells stably transfected with shFAM99A or shNC were detected by real-time qRT-PCR. 18S rRNA was used as an internal control. Data are shown as mean ± SD, student’s t-test (***P*<0.01, ****P*<0.001).


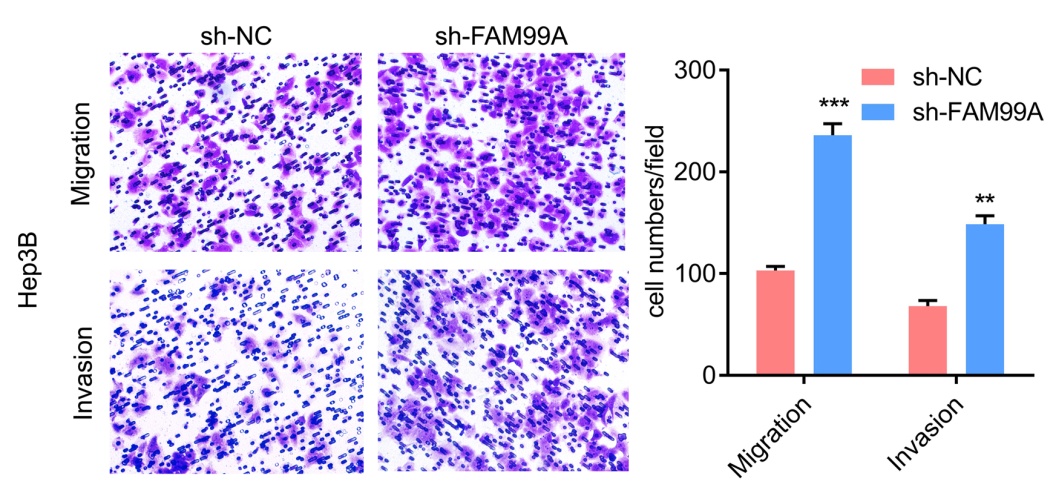

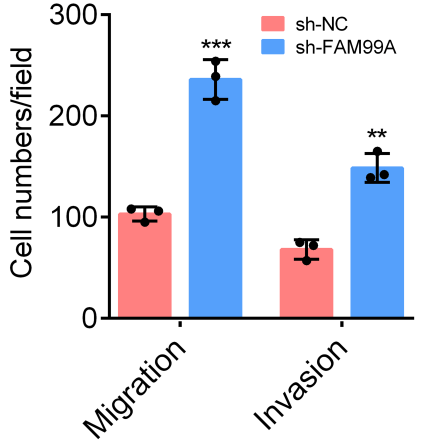


**Figure S5.** Representative and quantification results of transwell cell migration assay and invasion assay in Hep3B cells stably transfected with shFAM99A or shNC. Data are presented as mean ± SD; Student’s t-tests; ***P*<0.01, ****P*<0.001.

**
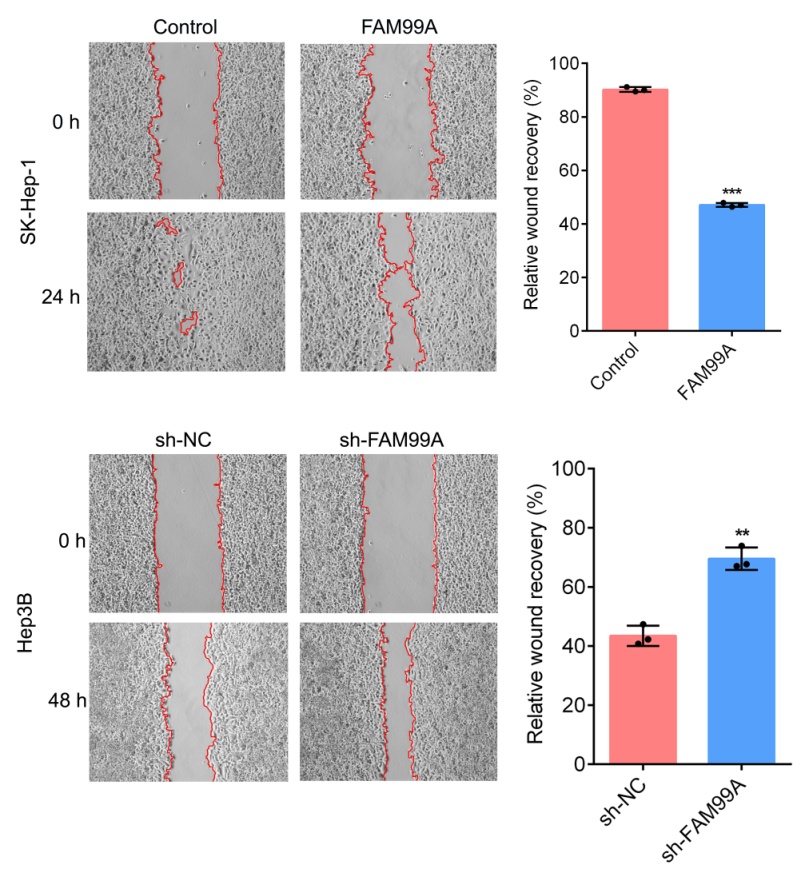
**

**Figure S6.** Representative and quantification results of wound healing assay. Data are presented as mean ± SD; Student’s t-tests; ***P*<0.01, ****P*<0.001.


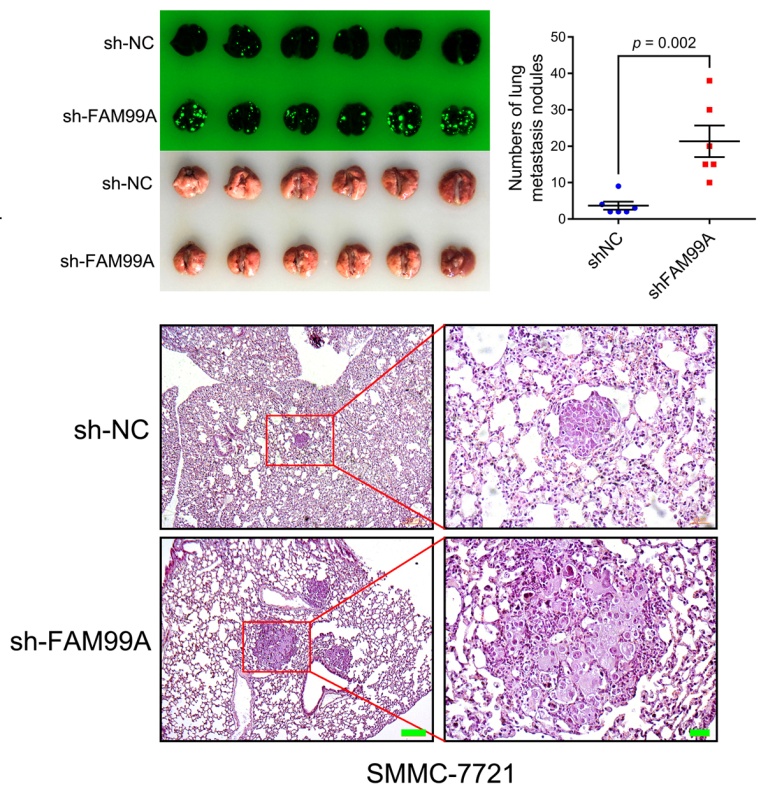


**Figure S7.** Representative images and quantification data of metastatic nodules in the lung tissues of mice. FAM99A knockdown SMMC-7721 cells were injected by tail vein in B-NDG mice. Upper left: macroscopic and fluorescent images. Upper right: quantification of lung metastatic nodules, data are presented as mean ± SD; Mann-Whitney U test. Lower: representative images of lung metastatic nodules stained with H&E (magnification, 50× and 200×; scale bar, 100μm and 20μm).


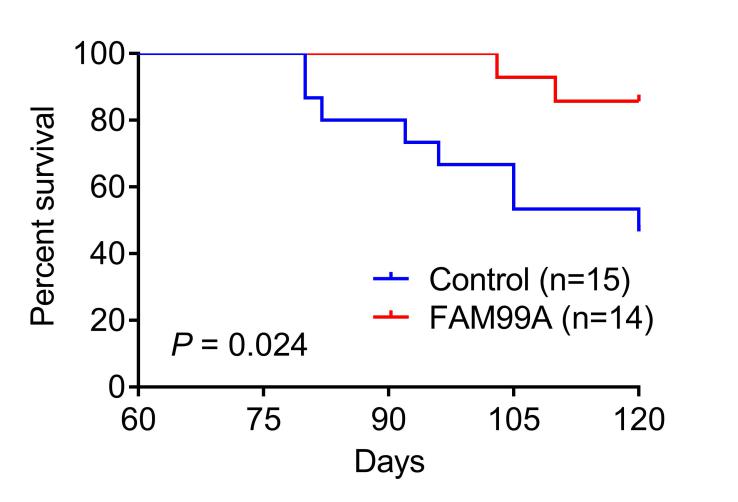


**Figure S8.** Survival analysis of mice tail vein implanted with SK-Hep-1 cells with FAM99A over-expression or not.





**Figure S9.** qRT-PCR analysis for FAM99A overexpression regulated miR-92a expression in SK-Hep-1 cells. *****P*<0.0001.


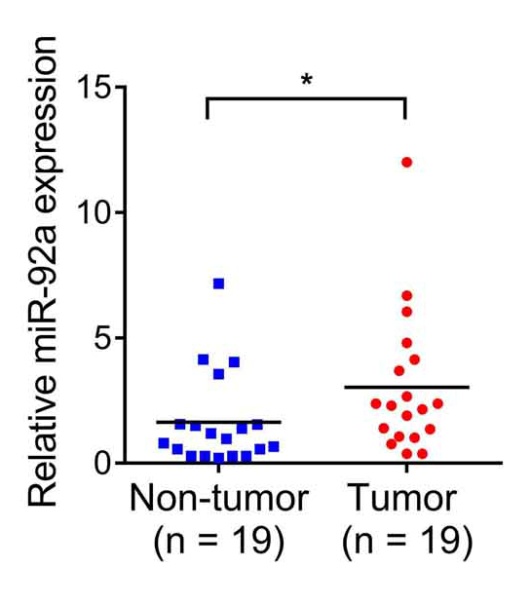


**Figure S10.** qRT-PCR analysis of miR-92a expression in 19 paired HCC tissues and adjacent para-tumor tissues. U6 was used as an internal control. Data are shown as mean ± SD, student’s t-test, **P*<0.05.


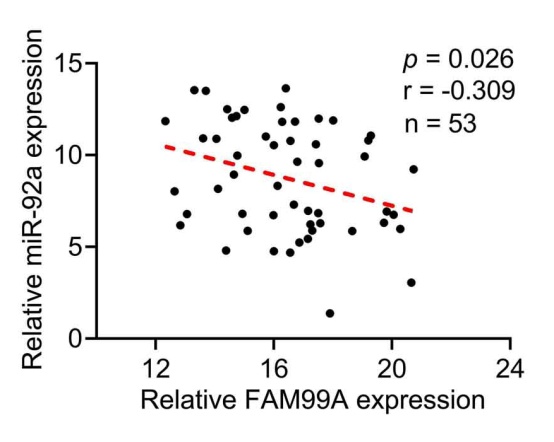


**Figure S11.** Pearson correlation analysis of FAM99A and miR-92a (r=-0.309, *P*=0.026, n=53).


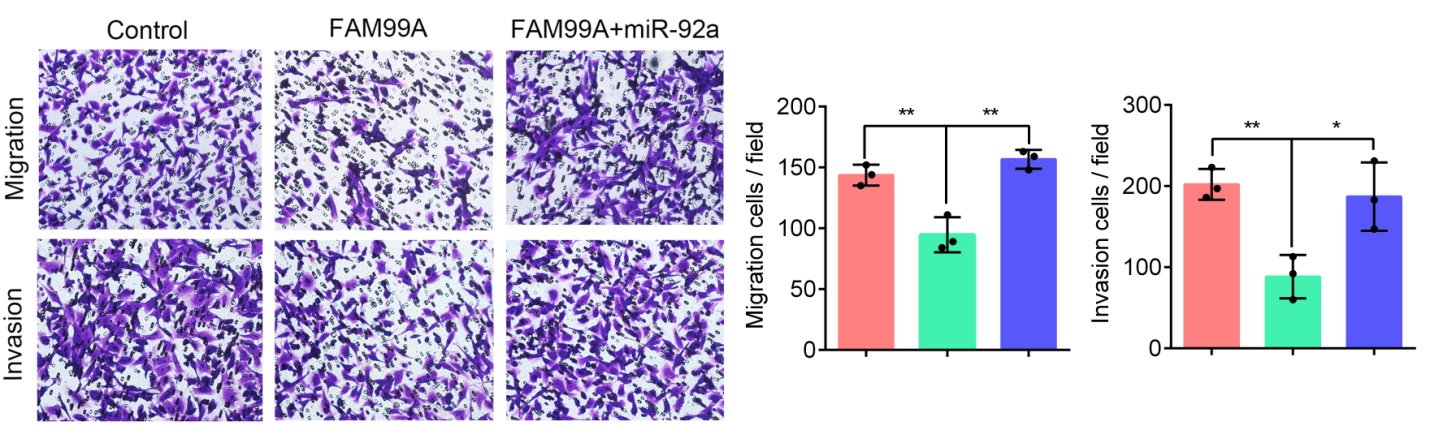


**Figure S12.** Representative images and quantification results of transwell cell migration and invasion assay in SK-Hep-1 cells stably transfected with FAM99A or co-transfected FAM99A and miR-92a. Data are presented as mean ± SD; Student’s t-tests, **P*<0.05, ***P*<0.01.


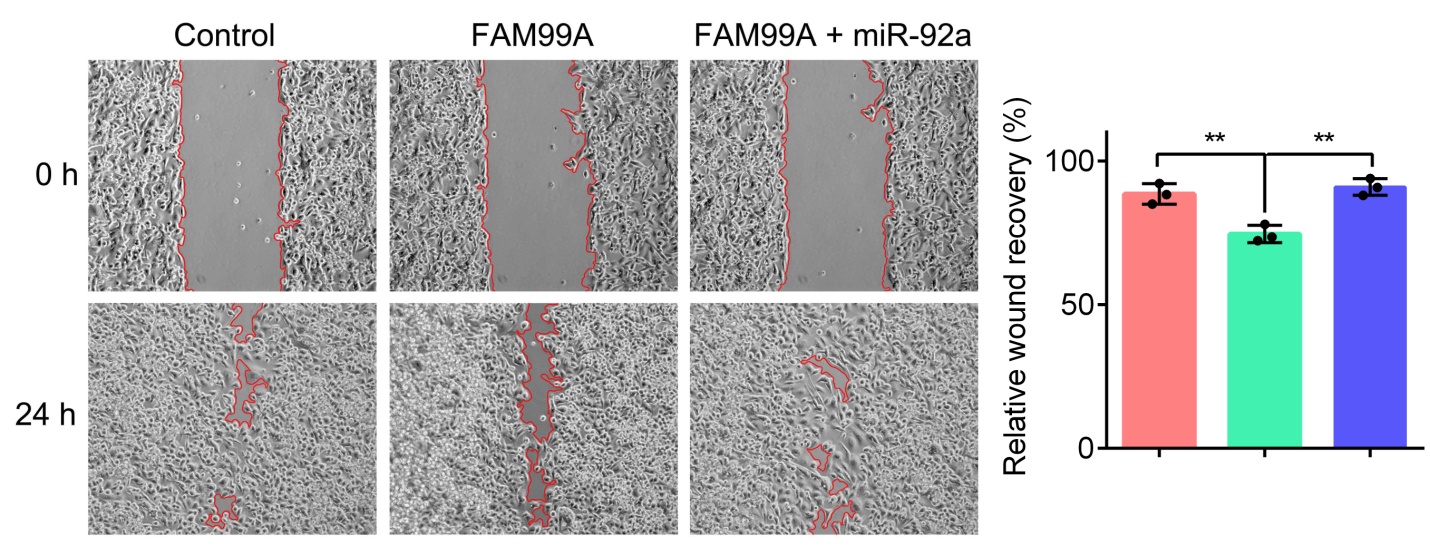


**Figure S13.** Representative images and quantification results of wound healing assay in SK-Hep-1 cells stably transfected with FAM99A or co-transfected FAM99A and miR-92a. Data are presented as mean ± SD; Student’s t-tests, ***P*<0.01.


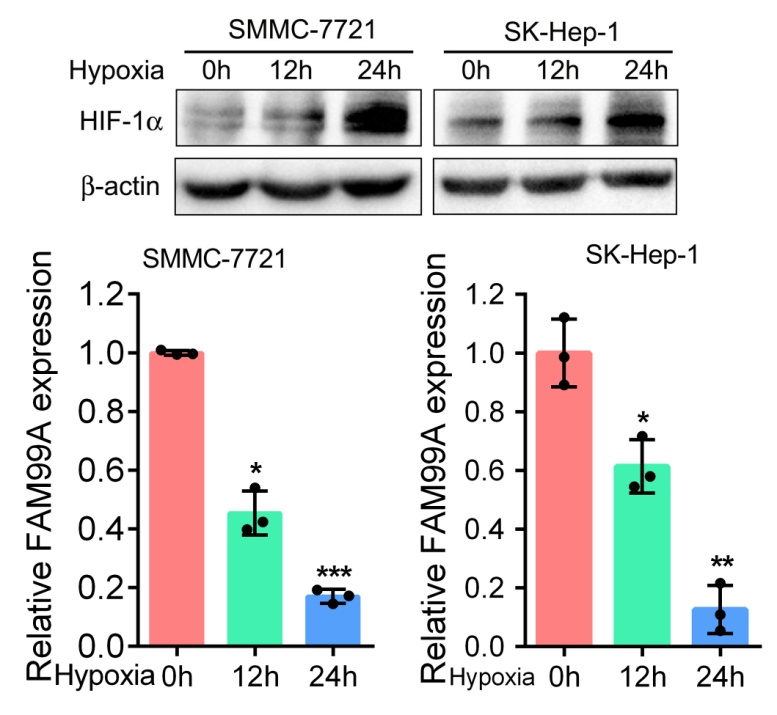


**Figure S14.** qRT-PCR detection of FAM99A expression under normoxic or different time of hypoxic conditions in SMMC-7721 or SK-Hep-1 cells. **P*<0.05, ***P*<0.01, ****P*<0.001.


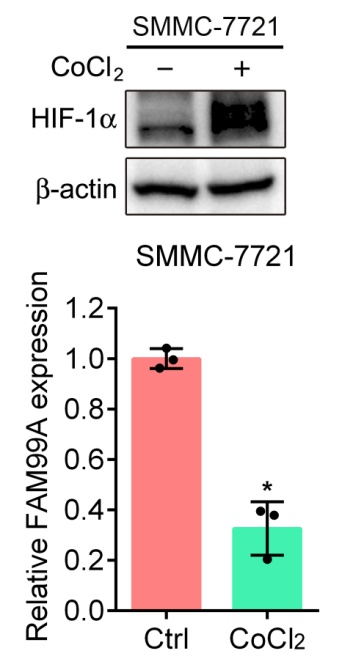


**Figure S15.** HIF-1α and FAM99A expression were detected by western blotting or qRT-PCR respectively after treatment with CoCl_2_ (100μm) for 24hr in SMMC-7721 cells. **P*<0.05.





**Figure S16.** Enrichment of HIF-1α binding HRE3 in normoxic or hypoxic conditions was detected by ChIP-qPCR. ***P*<0.01.

**
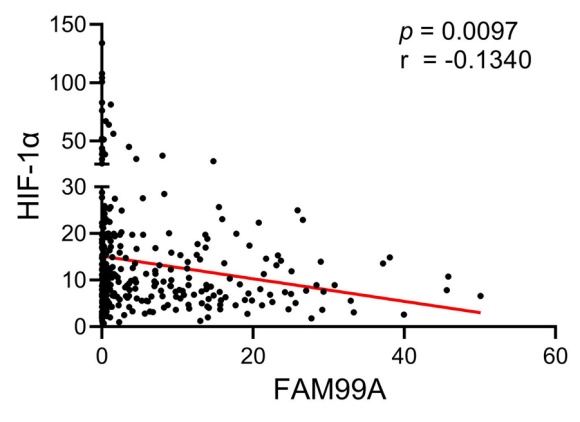
**

**Figure S17.** The correlation between HIF-1α mRNA level and FAM99A level in TCGA datasets. Data were subjected to Pearson correlation analysis (n=371, r=-0.1340, *P*=0.0097).


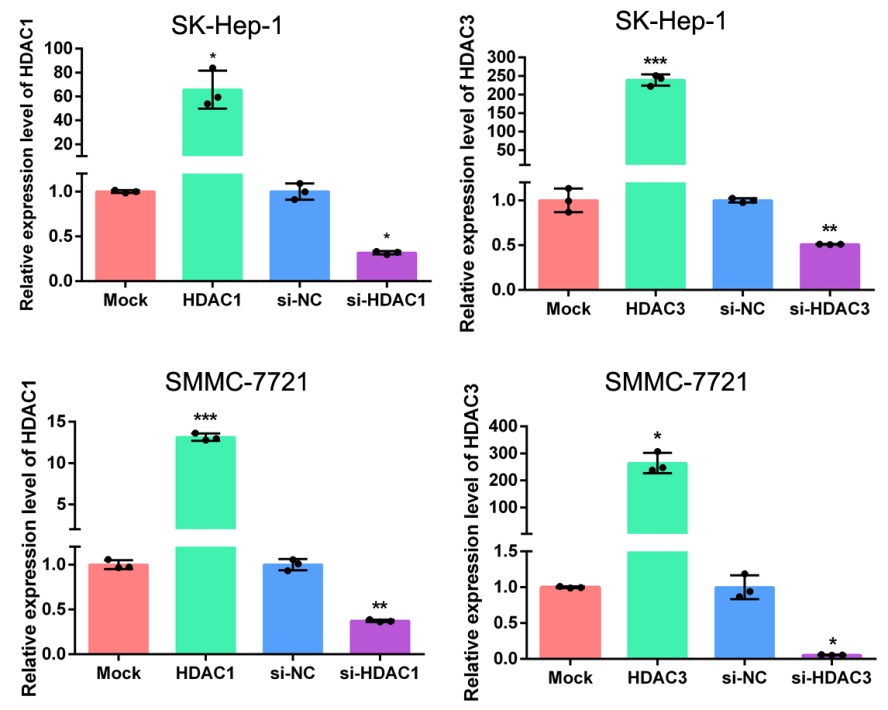


**Figure S18.** Verification of HDAC1/HDAC3 over-expression and knockdown in SK-Hep-1 and SMMC-7721 cells. SK-Hep-1 and SMMC-7721 cells were transfected with HDAC1/HDAC3 vector or si-HDAC1/HDAC3 respectively as indicated, the mRNA expression of HDAC1/HDAC3 were detected by real-time qRT-PCR. 18S rRNA was used as internal control. Data are shown as mean ± SD, student’s t-test, *P<0.05, ***P*<0.01, ****P*<0.001.


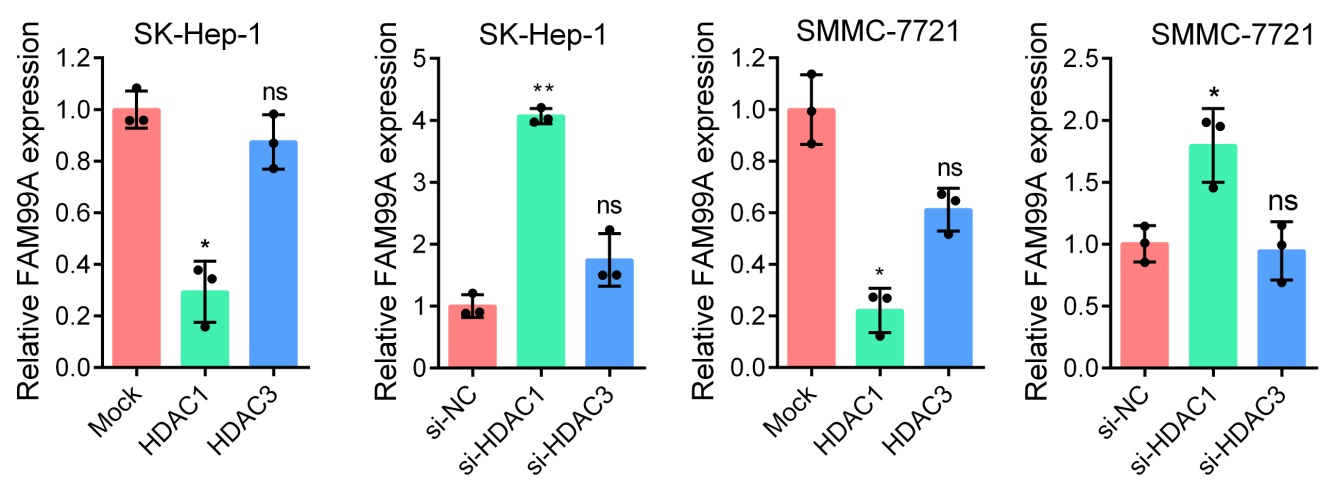


**Figure S19.** qRT-PCR analysis of FAM99A expression in SK-Hep-1 and SMMC-7721 cells transfected with HDAC1 and HDAC3 or siHDAC1 and siHDAC3. **P*<0.05, ***P*<0.01

**
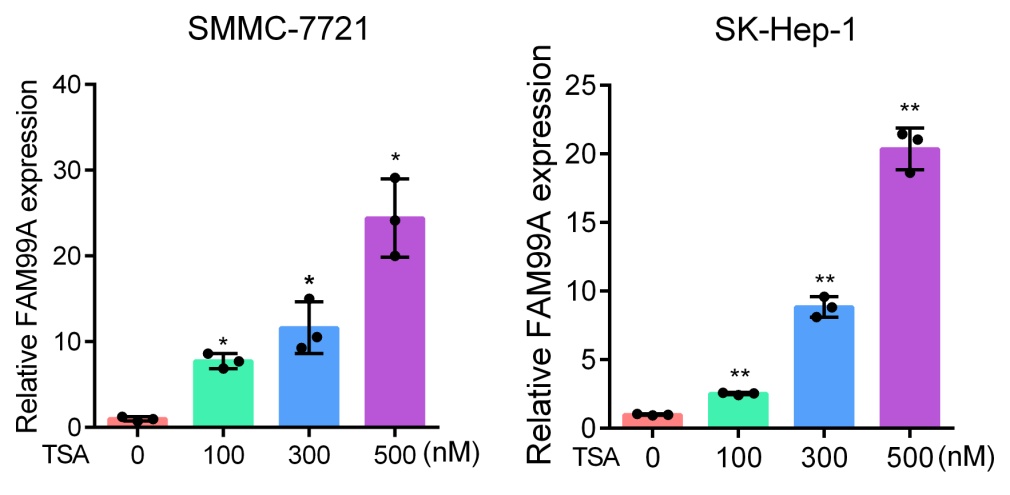
**

**Figure S20.** qRT-PCR detection of FAM99A expression in SMMC-7721 and SK-Hep-1 cells treated with TSA of different concentrations for 24hr. **P*<0.05, ***P*<0.01.





**Figure S21.** qRT-PCR analysis of FAM99A expression in SMMC-7721 cells cultured with or without different concentrations of TSA for 24hr under normoxic or hypoxic conditions. **P*<0.05, ***P*<0.01, *****P*<0.0001.

**
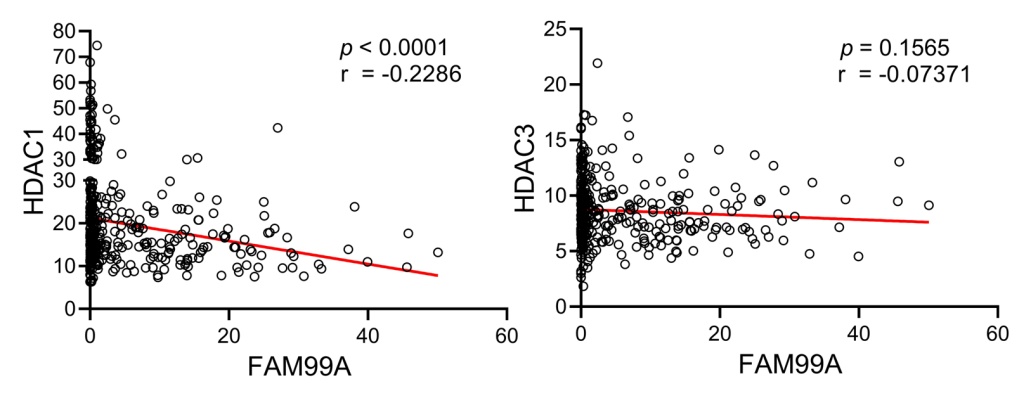
**

**Figure S22.** Pearson correlation analysis between FAM99A and HDAC1 (r=-0.2286, *P*<0.0001) or HDAC3 (r=-0.0737, *P*=0.1565) in TCGA datasets (n=371).





**Figure S23.** ChIP-qPCR analysis was conducted on the HRE3 using anti-HDAC1. The percent of enrichment was calculated relative to input. ***P*<0.01





**Figure S24.** ChIP-qPCR analysis was conducted on the HRE3 using anti-acetyl-histone H3 and H4. ****P*<0.001.


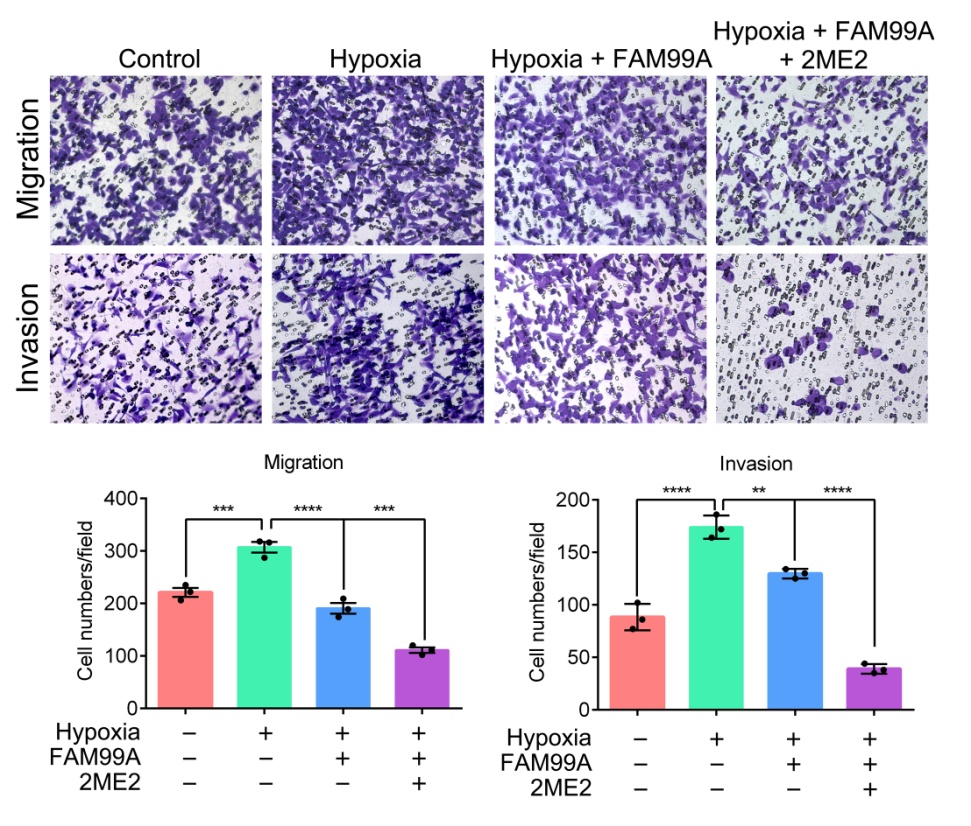


**Figure S25.** Representative images and quantification results of transwell cell migration and invasion assay in SK-Hep-1 cells under normoxic conditions, or transfected with FAM99A with and without 2ME-2 under hypoxic conditions. Data are presented as mean ± SD; Student’s t-tests; ***P*<0.01, ****P*<0.001, *****P*<0.0001.


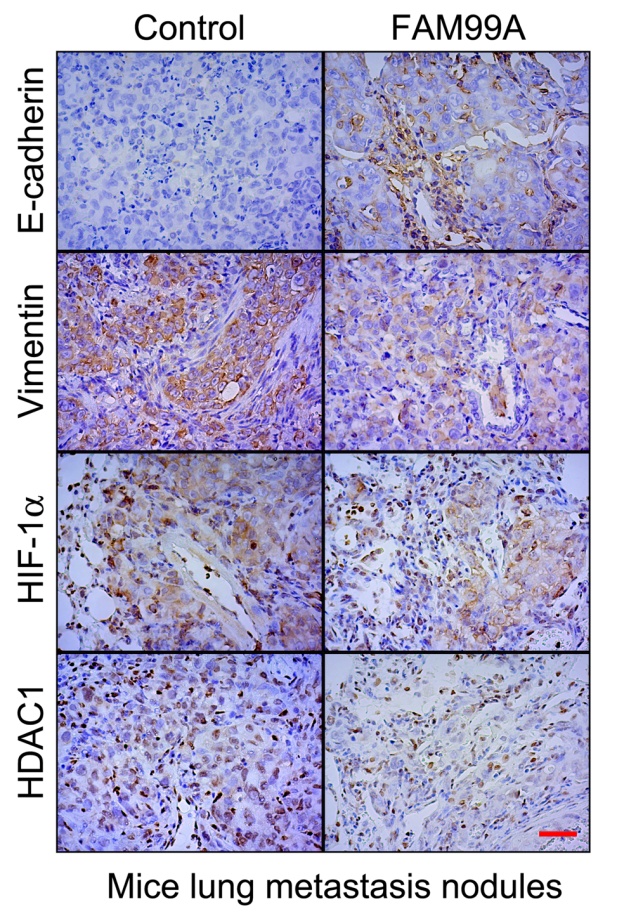


**Figure S26.** Representative images of IHC staining in lung metastasis nodules of mice (magnification, 200×; scale bar, 20μm).

Table S1.

Clinicopathological features of 103 HCC patients.

|  | | | | |
| --- | --- | --- | --- | --- |
| **Variables** | **Category** | **Low expression** | **High expression** | ***P* value** |
| Gender | Male | 45 | 44 | 1.000 |
|  | Female | 7 | 7 |  |
| Age (yrs) | >55 | 28 | 17 | **0.016** |
|  | ≤55 | 24 | 34 |  |
| Tumor size (cm) | >5 | 22 | 23 | 0.844 |
|  | ≤5 | 30 | 28 |  |
| Tumor number | >1 | 9 | 15 | 0.164 |
|  | 1 | 43 | 35 |  |
| TNM stage | III-IV | 9 | 10 | 0.804 |
|  | I-II | 43 | 41 |  |
| AFP (ng/mL) | ≥400 | 15 | 10 | 0.359 |
|  | <400 | 37 | 41 |  |
| HBV | Positive | 47 | 49 | 0.437 |
|  | Negative | 5 | 2 |  |
| Tumor capsule | Complete | 28 | 40 | **0.012** |
|  | Incomplete | 24 | 11 |  |
| Macrovascular invasion | Yes | 10 | 6 | 0.416 |
|  | No | 42 | 45 |  |
| Microvascular invasion | Yes | 24 | 26 | 0.695 |
|  | No | 28 | 25 |  |
| Hepatocirrhosis | Yes | 44 | 45 | 0.775 |
|  | No | 8 | 6 |  |
| Tumor differentiation | I-II | 10 | 25 | **0.002** |
|  | III-IV | 42 | 26 |  |
| Metastasis | Yes | 5 | 4 | 1.000 |
|  | No | 47 | 47 |  |
| Recurrence | Yes | 37 | 24 | **0.016** |
|  | No | 15 | 27 |  |

**Abbreviations:** TNM stage, Tumor-Node-Metastasis stage; AFP, alpha-fetoprotein.

Chi-square test was used to compare the statistical differences between the high expression and low expression of FAM99A. P <0.05 was considered as statistically significant.

Table S2.

Univariate and multivariate cox regression analysis affecting overall survival of HCC patients.

| **Variables** |  | **Case Number** | **HR (95% CI)** | ***P* value** |
| --- | --- | --- | --- | --- |
| **Univariate analysis** | |  |  |  |
| LncRNA-FAM99A (Low vs High) | | 52/51 | 0.415 (0.205-0.841) | **0.015** |
| Gender (F vs M) |  | 89/14 | 1.041 (0.406-2.673) | 0.933 |
| Age (> 55 vs ≤ 55yrs) | | 45/58 | 1.169 (0.612-2.232) | 0.636 |
| Tumor size (> 5 vs ≤ 5 cm) | | 45/58 | 2.624 (1.347-5.114) | **0.005** |
| Tumor number (> 1 vs 1) | | 63/40 | 1.283 (0.621-2.652) | 0.501 |
| TNM stage (III/IV vs I/II) | | 84/19 | 1.612 (0.760-3.416) | 0.213 |
| AFP (≥ 400 vs < 400 ng/ml) | | 25/78 | 2.019 (1.026-3.975) | **0.042** |
| Tumor capsule (Complete vs Incomplete) | | 68/35 | 0.683 (0.354-1.318) | 0.256 |
| Macrovascular invasion (Yes vs No) | | 16/87 | 2.720 (1.313-5.636) | **0.007** |
| Microvascular invasion (Yes vs No) | | 50/53 | 1.342 (0.702-2.565) | 0.374 |
| Hepatocirrhosis (Yes vs No) | | 89/14 | 0.713 (0.297-1.709) | 0.448 |
| HBV (Positive vs Negative) | | 96/7 | 0.747 (0.229-2.431) | 0.628 |
| Tumor differentiation (III-IV vs Ⅰ–Ⅱ) | | 68/35 | 0.461 (0.211-1.011) | 0.053 |
| Metastasis (Yes vs No) | | 9/94 | 3.013 (1.249-7.269) | **0.014** |
|  |  |  |  |  |
| **Multivariate analysis** | |  |  |  |
| LncRNA-FAM99A (Low vs High) | | 52/51 | 0.395 (0.187-0.833) | **0.015** |
| Tumor size (> 5 vs ≤ 5 cm) | | 45/58 | 2.226 (1.059-4.676) | **0.035** |

**Notes:** Hazard ratios and *P* values were analyzed by univariate and multivariate cox proportional hazards regression.

**Abbreviations:** TNM stage, Tumor-Node-Metastasis stage; AFP, alpha-fetoprotein; HR, hazard ratio; CI, confidence interval.

**Table S3.**

Primers used in real-time quantitative PCR

| Gene symbol | Sequence(5'→3‘） |
| --- | --- |
| FAM99A-F | TGTGGCTGTTTTGTGATGCG |
| FAM99A-R | GAGTGAGGGGTGCAGTTAGG |
| 18S rRNA-F | AGAAACGGCTACCACATCCA |
| 18S rRNA-R | CACCAGACTTGCCCTCCA |
| HDAC1-F | CATCGCTGTGAATTGGGCTG |
| HDAC1-R | ACCCTCTGGTGATACTTTAGCAG |
| HDAC3-F | CACCCGCATCGAGAATCAGA |
| HDAC3-R | AACTCATTGGGTGCCTCTGG |
| Actin-F | GAGAAAATCTGGCACCACACC |
| Actin-R | GGATAGCACAGCCTGGATAGCAA |
| HRE1-F | TTGTTTCCACCTTTGGGCTA |
| HRE1-R | ATCTGTTCCCAGCAAAACTCT |
| HRE2-F | CACAGTTCCGTGGCATT |
| HRE2-R | TGCAGTCACAATAGCCCAAA |
| HRE3-F | GCCCCACGAGCCATCAATTA |
| HRE3-R | CTCCCCTACTCTCCCCACAT |
